# Sentinel lymph node biopsy for breast cancer using methylene blue dye manifests a short learning curve among experienced surgeons: a prospective tabular cumulative sum (CUSUM) analysis

**DOI:** 10.1186/1471-2482-9-2

**Published:** 2009-01-27

**Authors:** Jeffrey M East, Christopher SP Valentine, Emil Kanchev, Garfield O Blake

**Affiliations:** 1 Department of Surgery, Cornwall Regional Hospital, Montego Bay, Jamaica; 2 Department of Surgery, Radiology, Anesthesia and Intensive Care, University of the West Indies, Mona, Kingston 6, Jamaica; 3 Department of Pathology, Cornwall Regional Hospital, Montego Bay, Jamaica

## Abstract

**Background:**

The benefits of sentinel lymph node biopsy (SLNB) for breast cancer patients with histologically negative axillary nodes, in whom axillary lymph node dissection (ALND) is thereby avoided, are now established. Low false negative rate, certainly with blue dye technique, mostly reflects the established high inherent accuracy of SLNB and low axillary nodal metastatic load (subject to patient selection). SLN identification rate is influenced by volume, injection site and choice of mapping agent, axillary nodal metastatic load, SLN location and skill at axillary dissection. Being more subject to technical failure, SLN identification seems to be a more reasonable variable for learning curve assessment than false negative rate.

Methylene blue is as good an SLN mapping agent as Isosulfan blue and is much cheaper. Addition of radio-colloid mapping to blue dye does not achieve a sufficiently higher identification rate to justify the cost. Methylene blue is therefore the agent of choice for SLN mapping in developing countries.

The American Society of Breast Surgeons recommends that, for competence, surgeons should perform 20 SLNB but admits that the learning curve with a standardized technique may be "much shorter". One appropriate remedy for this dilemma is to plot individual learning curves.

**Methods:**

Using methylene blue dye, experienced breast surgeons performed SLNB in selected patients with breast cancer (primary tumor < 5 cm and clinically negative ipsilateral axilla). Intraoperative assessment and completion ALND were performed for standardization on the first 13 of 24 cases. SLN identification was plotted for each surgeon on a tabular cumulative sum (CUSUM) chart with sequential probability ratio test (SPRT) limits based on a target identification rate of 85%.

**Results:**

The CUSUM plot crossed the SPRT limit line after 8 consecutive, positively identified SLN, signaling achievement of an acceptable level of competence.

**Conclusion:**

Tabular CUSUM charting, based on a justified choice of parameters, indicates that the learning curve for SLNB using methylene blue dye is completed after 8 consecutive, positively identified SLN. CUSUM charting may be used to plot individual learning curves for trainee surgeons by applying a proxy parameter for failure in the presence of a mentor (such as failed SLN identification within 15 minutes).

## Background

The benefits of sentinel lymph node biopsy (SLNB) in breast cancer patients with histologically negative axillary nodes, in whom axillary lymph node dissection (ALND) is thereby avoided, have now been well established [1]. The risks of lymphedema of the ipsilateral upper limb and distressing paraesthesiae and pain syndromes associated with ALND are reduced with consequential improved quality of life. SLNB is an accurate test of the metastatic status of axillary nodes [2], particularly when adjacent, palpably abnormal nodes are also reaped [3], with a false negative rate not exceeding 5% in properly selected patients [4] (those with primary breast tumors not exceeding 5 cm [5] and clinically negative axillae) and therefore, not surprisingly, has also been established as an oncologically safe and adequate procedure with disease free and overall survival similar to stage-matched patients having ALND [6-8].

Methylene blue dye is much cheaper than Isosulfan blue [9], does not cause hypersensitivity reactions [10] nor other significant complications (except skin necrosis [11], avoidable by meticulous injection technique when performing breast conserving surgery) and, most importantly, is as good as and possibly better at SLN mapping than Isosulfan blue [9,10,12-16]. Side effects such as blue discoloration of urine, stool and skin (does not cause tattooing) over the injection site are temporary and benign [17]. In North America and Europe, it is recommended that blue dye and radio-tracer mapping be combined as this approach may yield a higher SLN identification rate than blue dye alone [5,18-20], although some studies have not confirmed this advantage [12,16,21]. Radio-tracer mapping is very expensive (prohibitively so in most developing countries), cumbersome (requiring time-consuming preoperative preparation and increased operating time), has no significant SLN detection advantage as single agent over blue dye [12] (except detection of internal mammary SLN, an uncommon finding of doubtful clinical and therapeutic significance) and may pose radiation risk to pathologists handling the nodes [22]. Combination of the two techniques achieves a range of 0% to 18% increase in SLN identification rate over blue dye alone [5,12,16,18-21]. Since 60% of clinically negative axillae are also pathologically negative [23], combining the radio-labeled tracer technique with the blue dye technique stands to benefit only an additional 0 to 11 per 100 women (by way of avoidance of ALND). This seems to be an unjustifiably high price to pay for such a small additional benefit, especially since SLNB is not a therapeutic procedure and does not offer any survival advantage to patients. Methylene blue dye as a single agent is therefore well suited to enable surgeons in developing countries to offer the important technique of SLNB without significantly compromising the quality of the test.

The American Society of Breast Surgeons recommends that, for competence, surgeons should perform 20 SLNB procedures either under supervised mentoring by an experienced colleague or followed immediately by completion ALND [4,24]. It is also recommended that the false negative rate not exceed 5% and the SLN identification rate not be less than 85%. Low false negative rate is a reflection of the high inherent accuracy with which the sentinel node reflects the metastatic status of axillary nodes, now an established fact [2], as well as a function of selection of patients for the procedure who are less likely to harbor lymph node metastases. SLN identification rate is, like false negative rate, subject to axillary nodal metastatic load (patient selection) but also to injection site, volume and choice of mapping agent, location of the SLN, and, importantly, the surgeon's skill at dissecting the axilla. Being more susceptible to technical failure, SLN identification therefore seems to be a more reasonable target for learning curve analysis than false negative rate. Others agree that SLN identification should be the objective of the learning process, especially since failure to find the SLN does not have therapeutically deleterious consequences, ALND being performed in those cases [25].

The American Society of Breast Surgeons admits that the learning curve for surgeons using a standardized technique may be "much shorter" than 20 cases [24] but does not suggest a remedy by way of a reliable method for assessing individual learning curves. It should hardly be surprising that experienced breast surgeons can be expected to master this technique after just a few cases since, being skilled at ALND, they know how to find the nodes draining the breast and should therefore have little difficulty locating a blue sentinel node (radio-tracer mapping adds another level of difficulty), as long as patients are properly selected and the SLN resides within levels I and II of the axilla. Clearly a method is needed for plotting learning curves with the capacity to test and predict individual performance with reference to a standardized level of competence.

Cumulative sum (CUSUM) charting is one of a group of statistical process control methods used by engineers, particularly in manufacturing, to monitor quality of output and, with boundary limits set by the sequential probability ratio test (SPRT), to indicate when a process is out of control [26]. Statistical process control methods are now being used in medicine predominantly for quality control monitoring [27-29] but more recently, for learning curve analysis [30-34]. CUSUM plots are an excellent method for determining learning curves for any procedure with output variables that can be dichotomized and hold the promise of individualizing credentialing and competency certification requirements and ending what has been called the tyranny of mandatory case numbers.

CUSUM analysis has been applied to the learning curve for SLNB as a retrospective quality control tool [25] but not for prospective learning curve analysis. Other learning curve methodologies have also been applied for SLNB [35]. In this study, using methylene blue dye as the mapping agent, tabular CUSUM control charting is used to prospectively evaluate the learning curve of individual surgeons for SLNB and signal achievement of an acceptable, predetermined level of competence.

## Methods

The study was approved by the Ethics Committee of the Ministry of Health of Jamaica. Informed consent was received from all participating patients. All patients of either gender presenting to the Cornwall Regional Hospital (CRH) with breast cancer after implementation of the study protocol were assessed for eligibility. Participants were prospectively selected for SLNB if they had an established diagnosis of adenocarcinoma of the breast, a Tis, T1 or T2 primary breast tumor (ie, not exceeding 5 cm in greatest dimension) and a clinically normal ipsilateral axilla. Completion ALND was performed on all participants up to and just beyond the end of the learning curves of both surgeons (13 cases total) as the authors felt that, this being the first such study in Jamaica, definitive treatment of the axilla during this phase should not differ from the pre-existing standard of care (a commitment also given to the Ethics Committee). Subsequent patients were to have immediate ALND only if the SLN was positive on intraoperative assessment or could not be identified, and delayed ALND if an initially negative SLN turned out to be positive on paraffin section.

After induction of anesthesia, 5 cc of 1% methylene blue for injection is infiltrated into the subareolar tissue (yields higher identification rate with blue dye than other sites [36-38]), except when there is a transverse biopsy scar across the upper outer quadrant or axillary tail of the breast, in which case the dye is injected into the parenchyma superolateral to the scar (the presence of such a scar reduces SLN identification rate after subareolar injection [36,37]). In either case, special care must be taken to avoid injection into the skin or submammary connective tissue and muscle. The breast is then massaged for 5 minutes.

The surgical site is prepared and the surgeon goes directly for the SLN via an axillary incision if breast conserving surgery is the definitive procedure. If mastectomy is the definitive procedure, the surgeon goes for the sentinel node after creating the superior flap. All blue nodes and any node receiving a blue lymphatic channel are sentinel nodes. After removal of sentinel nodes, adjacent tissue is palpated and any additional hard or large nodes are also removed. Total number of nodes removed should usually not exceed three, otherwise the benefits of the limited dissection required for SLNB could be compromised. The following variables are then recorded unto a pre-coded form: surgeons (surgeon and first assistant), mastectomy versus breast conserving surgery, site of injection, SLN identified or not, number of SLNs, Berg's level at which SLN found, and number of any non-SLN removed. The unfixed nodes are sent to the pathologist. The operation is then completed, including ALND (in the first 13 cases and if indicated in the others).

The technique for intraoperative processing of the sentinel node for touch imprint cytology differed between the 2 pathologists. One pathologist divides the nodes in halves longitudinally, whilst the other slices them into 2 to 3 mm transverse sections. The former stains cytology specimen separately by May Grunwald-Giemsa, Diff-Quik and haematoxylin eosin (H&E) in a shortened regimen and the latter uses only the H&E technique. Both pathologists used a similar processing technique and rapid H&E staining for frozen section. Although the protocol called for performance of touch imprint cytology and frozen section on all sentinel nodes, the latter procedure was not always possible, usually because of equipment failure.

The following variables are recorded by the pathologist on the original form: SLN cytology (positive or negative), SLN frozen section, SLN paraffin sections and ALND paraffin sections. All variables were entered into a database in STATA version 8 for analysis.

## Results

Tables 1 and 2 show the results of the 24 cases performed by the authors. All patients were female. SLNs were identified in all of the first 16 consecutive cases and in 6 of the subsequent 8. In 68% (15/22), only one SLN was identified. Additional non-sentinel nodes were reaped in 27% (6/22). Only 1/29 SLN was identified at Berg's level II, the rest being found at level I. One surgeon (JE) failed to identify SLN in his 14^th ^and 16^th ^cases. Both had more than 3 nodes involved by metastatic disease in the ALND specimen. There were no surgical false negatives among the 13 patients having completion ALND. Of 7 positive paraffin-embedded SLN in this group, three (43%) were negative by touch-imprint cytology (and frozen section in one case). There were no other cytological false negatives among the subsequent 9/11 cases in which SLN were identified. In 57% (4/7) of cases with paraffin-positive SLN, the SLN was the only node involved by metastatic disease.

**Table 1:** Profile and results of SLNB and completion ALND.

**Case** **No**.	**Surgeons**	**Breast** **Surgery**	**Inj**. **Site**	**No. SLN** **ident**.	**Berg's** **Level**	**Other** **Nodes**	**TIC**	**FS**	**SLN** **PARA**	**ALND**
1	JE/CV	BCS	SubA	1	I	0	+	+	+	-
2	JE/CV	BCS	SubA	1	I	1	-	-	+	-
3	JE/CV	BCS	SubA	2	I	0	+	+	+	+
4	JE	Mastect	SubA	1	I	0	+	+	+	+
5	JE/CV	BCS	SubA	1	I	0	-	ND	-	-
6	JE	BCS	SubA	1	I	0	-	ND	-	-
7	JE/CV	Mastect	SubA	1	I	0	-	-	-	-
8	CV	Mastect	SubA	1	I	1	-	ND	+	+
9	JE	Mastect	IntraP	2	I	0	-	ND	-	-
10	JE	Mastect	SubA	1	II	2	-	ND	-	-
11	CV	Mastect	SubA	1	I	1	-	ND	+	-
12	CV	Mastect	SubA	1	I	0	+	ND	+	-
13	JE	BCS	SubA	2	I	0	-	ND	-	-

BCS – Breast conserving surgery; Mastect – Mastectomy; SubA – subareola; IntraP – Intraparenchymal; TIC – Touch imprint cytology; FS – Frozen section; SLN PARA – SLN paraffin sections; ALND – ALND paraffin sections; ND – not done; NA – not applicable

**Table 2:** Profile and results of SLNB with ALND only if SLN +ve or not found.

**Case** **No**.	**Surgeons**	**Breast** **Surgery**	**Inj**. **Site**	**No. SLN** **ident**.	**Berg's** **Level**	**Other** **Nodes**	**TIC**	**FS**	**SLN** **PARA**	**ALND**
14	JE	Mastect	SubA	2	I	0	-	ND	-	ND
15	JE	Mastect	SubA	2	I	1	-	ND	-	ND
16	JE	Mastect	SubA	1	I	0	-	ND	-	ND
17	JE	Mastect	SubA	0	NA	0	NA	NA	NA	+
18	CV	Mastect	SubA	1	I	0	-	ND	-	ND
19	JE	Mastect	IntraP	1	I	0	-	ND	-	ND
20	JE	Mastect	IntraP	0	NA	0	NA	NA	NA	+
21	JE	Mastect	SubA	2	I	0	-	ND	-	ND
22	CV	Mastect	SubA	2	I	0	-	ND	-	ND
23	JE	Mastect	SubA	1	I	0	-	ND	-	ND
24	CV	Mastect	SubA	1	I	1	-	ND	-	ND

Legend as for Table 1.

Only one patient (case number 3) suffered a significant complication, with dehiscence and delayed healing of that part of a partial mastectomy wound which extended into the peri-areolar region into which methylene blue had been injected. The wound healed in time for the patient to start adjuvant chemotherapy one month after surgery.

**Figure 1:**
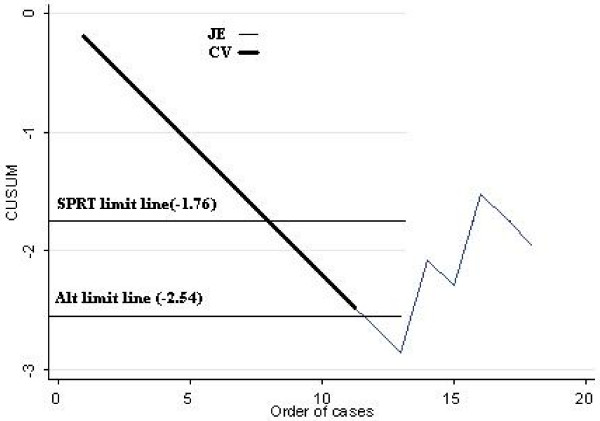
**CUSUM plots for two surgeons (superimposed for the first 11 cases)**. Primary SPRT limit line is crossed after 8 consecutive, positively identified SLN and alternate line is crossed after 12. Parameters for the primary line are from Table 3. and are similar for the alternate line except for a type 2 error of 0.1 instead of 0.2. That part of the plot beyond either lower SPRT limit line is statistically meaningless but is left in place to allow for drawing of other hypothetical limit lines. In practice, the plot may be restarted at 0 after the SPRT line is crossed, for the purpose of process monitoring.

The cumulative sum formula (Table 3) was applied to each surgeon's results and used to create their individual plots (Figure 1). A sequential probability ratio test (SPRT) for binary variables [26] was used to calculate upper and lower limit values, assuming a type 1 error (α) of 0.05, a type 2 error (β) of 0.2, an acceptable SLN identification failure rate of 15% and an unacceptable failure rate of 30% (Table 3). The plots of the two surgeons in Figure 1. are superimposed for 11 cases. Note that the lower SPRT limit line, based on parameters from Table 3., is intersected after 8 consecutive cases in which the sentinel node was identified, predicting that there is no statistically significant difference between the identification rate of the surgeons who achieved this number and the 85% identification rate recommended by the American Society of Breast Surgeons for the learning phase. An alternate limit line, based on a type 2 error of 0.1 rather than 0.2 (all other parameters are the same) is crossed after 12 consecutive cases. The upper limit line is omitted from Figure 1. as it has no relevance to the interpretation of this chart.

**Table 3:** CUSUM and SPRT calculations, according to Davies et al [26].

p_0 _is the acceptable failure rate for SLN identification (0.15)
p_1 _is the unacceptable failure rate (set at 0.3)
α is the type 1 error (set at 0.05)
β is the type 2 error (set at 0.2)
The CUSUM is increased by 1 - s for a failure and decreased by s for a success.
a = ln [(1 - β)/α] = 2.773
b = ln [(1 - α)/β] = 1.558
P = ln(p_1_/p_0_) = 0.693
Q = ln [(1 - p_0_)/(1 - p_1_)] = 0.194
s = Q/(P + Q) = 0.219
h_0_, the lower boundary limit, = -b/(P + Q) = -1.756
h_1_, the upper boundary limit, = a/(P + Q) = 3.126

## Discussion

The unavailability of frozen section for the majority (19) of cases is not a major shortcoming as touch imprint cytology is as accurate for intraoperative lymph node assessment [39], although both methods are limited in accuracy compared to paraffin sections [40], as confirmed herein. Insistence on frozen section would also limit the portability of SLNB to less well equipped hospitals. Successful application of ambulatory SLNB [41] with examination of paraffin-embedded sections and delayed definitive breast surgery offers improved accuracy of SLN diagnosis as well as the possibility of performing this technique even in hospitals without a resident pathologist. The obvious disadvantage of this latter approach is that all patients end up with 2 operations instead of only the small minority with pathological false negative intraoperative node assessment who require delayed ALND.

The presence of metastatic disease in several axillary nodes of both cases in which no SLN was found implies that they were poorly selected to begin with and that failure to identify was probably due to failure of the SLN to take up the dye [42] rather than failure of technique. Routine preoperative sonographic assessment of axillary nodes has the potential to reduce SLN identification failure for this reason if SLNB is restricted to sonographically negative rather than clinically negative cases [43].

The case of wound dehiscence was likely due to injection of dye into or too close to the skin and can be avoided by careful injection into the central sub-areolar tissue.

There are several formulations of CUSUM charting applicable to learning curve analysis. The simple CUSUM [34], also called O-E (observed – expected) CUSUM chart, is based on the formula: S_n _= Σ(X_o _- X_i_)

where S_n _is the cumulative sum, X_o _is the target competence level (expressed as a decimal fraction) and X_i _= 1 for a successful outcome and 0 for a failure. Therefore for an individual successful attempt, the CUSUM decreases by X_o _- 1 and for a failure, it increases by X_o_. When the curve changes its gradient from upwards through horizontal to decreasing, the learning curve is considered complete [34]. This is useful for charting learning curves for procedures which take some time to master (that is, for procedures with a high failure rate early on). In charting procedures with a high success rate early on, as in this study, the simple CUSUM leads to the conclusion that there is no learning curve [34] as the plot starts in a negative direction (the direction of success) and maintains its gradient.

The tabular CUSUM (also called SPRT chart and log-likelihood CUSUM) is in effect a graphical representation of a hypothesis test comparing each sequential occurrence of a binary outcome variable to upper and lower limit values [26,27,31,32]. The test is effectively re-performed for each event in the sequence, thereby taking into account cumulative past performance, as reflected by the candidate's position on the plot just prior to the next event. In this formulation, the CUSUM decreases by s for a success and increases by 1 - s for a failure (see Table 3. for calculation of s). The null hypothesis for this test states that, for a given occurrence, the true failure rate is not different from the target failure rate. When the plot crosses the lower limit line, the null hypothesis cannot be rejected and this signals that the predetermined target level of competence has been achieved. If the plot crosses the upper limit line, it must either change gradient (as for the simple CUSUM) or return to a trailing lower limit line placed at a distance below the upper line equal to the distance from the X-axis to the original lower limit line, before the learning curve is considered complete [31]. In a recent variation of the tabular CUSUM, the plot is not allowed to rise above the X-axis and the learner is therefore punished less severely for an early sequence of failures [44]. Limit lines may be calculated using the method advocated by Davies et al [26], as was done in this study, or the log-likelihood method advocated by Wald [28].

For complex procedures, the score for each attempt is risk-adjusted by multivariable regression and then applied to an SPRT CUSUM chart [30,45,46]. Calculation of SPRT limits in these risk-adjusted CUSUM charts is more complex than in the preceding type of chart.

The output of formulas for the tabular CUSUM are subject to manipulation, and therefore the values for acceptable failure rate, unacceptable failure rate, type 1 error and type 2 error must be chosen carefully, although subjectivity cannot be completely avoided. Acceptable and unacceptable failure rates are usually set by consensus or convention. Acceptable failure rate should not be set so low that only a few gifted surgeons with perfect patient selection can achieve it. Unacceptable failure rate should be set at a level above which either the effectiveness (for procedures without serious consequences) is below average or the risk (for procedures with serious consequences) above average. Type 1 error should be set low (maximum 0.1) if we do not wish to falsely identify a competent surgeon as being incompetent [47] (that is, falsely assuming that there is a difference from the target value when none exists). Conversely, type 2 error should be set low if we worry about falsely identifying an incompetent surgeon as competent (that is, falsely assuming that no difference exists from the target value when one exists). In practice, type 2 error should not be set too low if the procedure is easy to learn and if the consequences of failure are not serious.

Table 4. indicates the different numbers of consecutively successful cases required to cross the lower SPRT limit line for different combinations of acceptable and unacceptable failure rates and type 1 and type 2 error. It also indicates the total number of cases required to cross the limit line after 3 failures (the effect of failures on the number of cases required to cross the limit line is independent of where they occur), the number allowed by the American Society of Breast Surgeons in their recommended 20 cases. For a technically more difficult operation or for procedures with more serious consequences for failure than those for failure to identify a SLN, it could be successfully argued that both β and p_1 _should be set lower than 0.2 and 0.3 respectively. Given that SLNB using methylene blue dye is an easy operation and that the consequence of failure to identify the SLN simply results in a possibly unnecessary ALND, which is still the standard of care, we do not believe that our choice of 0.2 for β is too high. Studies have reported SLN identification failure rates with blue dye of 0.25 [5] and 0.27,[48], and therefore, we also believe that our choice of unacceptable failure rate of 0.3 is reasonable. Conversely, although the literature suggests that SLN identification rate can be as high as 95% and even higher, it would be a mistake to set the acceptable failure rate for the learning curve at 0.05 since this assumes perfect selection of patients and perfect technique.

**Table 4:** Number of consecutive and total cases required to cross the lower limit line for different combinations of acceptable (p_0_) and unacceptable (p_1_) failure rates, type 1 error (α) and type 2 error (β).

**Total no. cases, assuming 3 failures**	**No. consecutive cases, assuming no failures**	**p_0_/p_1_**	**α/β**
97	55	0.05/0.1	0.05/0.05
84	42	"	0.05/0.1
46	29	"	0.05/0.2
49	27	0.1/0.2	0.05/0.05
43	21	"	0.05/0.1
36	13	"	0.05/0.2
29	15	0.15/0.3	0.05/0.05
27	12	"	0.05/0.1
22	8	"	0.05/0.2

**Figure 2:**
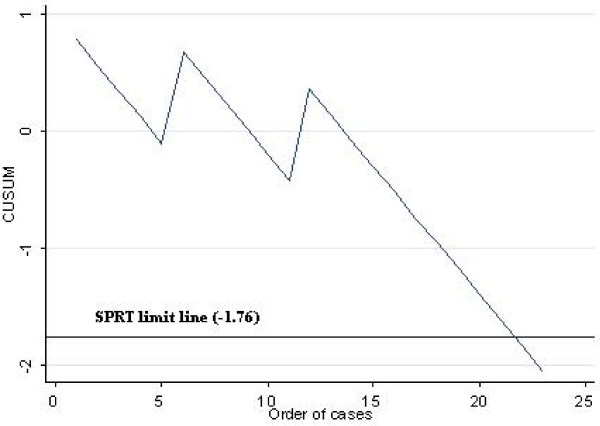
**Hypothetical CUSUM plot for surgeon/trainee failing to identify 1^st^, 6^th ^and 12^th ^SLN**. Parameters are from Table 3. Plot crosses SPRT limit line after 22 cases.

The tabular CUSUM applied herein is excellent for plotting the learning curve of trainee surgeons being taught to perform SLNB. All that is required is a proxy measure for failure to identify the SLN in the presence of a mentor, such as failure to find the SLN within a specified time, say 15 minutes. Figure 2. (see also Table 4.) is an illustration of how the CUSUM plot behaves in the hypothetical scenario where the trainee surgeon fails to identify sentinel nodes in the 1^st^, 6^th ^and 12^th ^cases. Applying the parameters from Table 3, 22 cases would then be required to cross the lower limit line and signal the end of the learning curve, a number marginally higher than the 20 cases recommended by the American Society of Breast Surgeons for a similarly allowed failure rate of 3 cases. This latter observation provides further support for the reasonableness of our choice of parameters.

## Conclusion

Methylene blue dye is an accurate and cost-effective single agent for sentinel lymph node mapping in breast cancer. Sentinel node identification rate is a more reasonable target for assessment of learning curves of surgeons than is false negative rate. Application of a tabular CUSUM chart, with SPRT limit values based on a target identification rate of 85%, and a reasonable choice of other parameters, demonstrates that experienced breast surgeons have completed the SLNB learning curve after 8 consecutive successful attempts using methylene blue. This type of learning curve analysis can be readily applied to trainee surgeons by using a proxy measure for failure in the presence of a mentor, such as failure to identify the SLN within 15 minutes. Using the same parameters, a trainee surgeon allowed 3 failures requires 22 cases to complete the learning curve.

## Competing interests

The authors declare that they have no competing interests.

## Authors' contributions

All authors participated in the acquisition of data and revision of the manuscript. JME and CSPV performed sentinel node biopsy whilst EK and GOB performed pathological assessment of the lymph nodes. JME conceived of the study, determined the design, performed the statistical analysis, interpreted the data and drafted the manuscript, with specific inputs from EK and GOB to the methods section with respect to their techniques for intraoperative pathological analysis of sentinel nodes. All authors read and gave final approval of the version submitted for publication.

## Pre-publication history

The pre-publication history for this paper can be accessed here:


